# Age-moderating effect in prepotent response inhibition in boys with Asperger syndrome: a 2.5 years longitudinal study

**DOI:** 10.1007/s00406-018-0915-1

**Published:** 2018-06-25

**Authors:** Elisabeth M. Weiss, Claudia Walter, Andreas Fink, Günter Schulter, Erich Mittenecker, Ilona Papousek

**Affiliations:** Department of Psychology, Biological Psychology Unit, University of Graz, Univ.-Platz 2, 8010 Graz, Austria

**Keywords:** Autism spectrum disorder, Longitudinal, Mittenecker pointing test, Cognitive flexibility, Inhibition, Memory updating

## Abstract

Following our previous cross-sectional analysis, indicating age-related improvements of response inhibition in a random-motor-generation task (MPT) in adolescents with Asperger syndrome (AS), the present study reports data from a 2.5-year follow-up examination in the original sample. We found more marked improvements within the follow-up interval in younger AS children, while older AS boys as well as typically developing (TD) boys remained at a relatively constant level throughout. The current longitudinal study further substantiates the notion that AS children (on average) catch up with TD children when they grow older as regards the basic inhibition of developing routine response patterns.

## Introduction

Neurocognitive dysfunction and especially deficits in specific executive domains are highly prevalent in autism spectrum disorder (ASD) and play an important role in the core problems of this pervasive developmental disorder, including difficulties in social interaction and communication as well as restricted and repetitive behaviors. In a recent meta-analysis involving a large number of ASD individuals, a moderate overall effect for impaired executive functions (EFs) was found that was relatively stable across different age groups [1]. Furthermore, no specific EF pattern unique to individuals diagnosed with ASD was identified across different EF subdomains (concept formation, mental flexibility, fluency, planning, response inhibition, working memory).

Maturational changes of the prefrontal cortex from childhood to adolescence result in marked improvements in EFs as children age [2, 3]. Despite quite extensive literature on executive dysfunction in ASD, relatively little is known about the developmental trajectories in ASD, and especially long-term follow-up studies are scarce. Up to now, the rare studies with a longitudinal design show a substantial variability in the growth trajectories of autistic children’s executive functions (for a review see [4]).

Previously we reported cross-sectional data on a sample of 23 boys with Asperger syndrome (AS) and 23 matched healthy controls using a random-motor-generation task that examined two components of cognitive flexibility (inhibition of prepotent responses and memory monitoring/updating) [5]. We found poorer inhibition and more repetitive response patterns only in young children diagnosed with AS but no such group differences between AS children and typically developing (TD) children (independently of age) in memory monitoring and updating. Following our previous cross-sectional analysis, indicating age-related improvements of response inhibition in AS adolescents, the present study reports data from a 2.5-year follow-up examination in the original sample.

## Materials and methods

### Participants and procedure

Thirty children from the initial study (*n* = 46) were available for further testing [15 boys with AS (*M* = 11.2 ± 2.8 years) and 15 TD boys (*M* = 12.2 ± 2.6 years; *t*(28) = 1.055, *p* = 0.30)]. Diagnostic criteria of AS conformed to ICD-10 (F84.5; DIMDI [6]), as diagnosed by a child psychiatrist in the initial study. Seven boys of the AS group (none of the TD group) had an additional diagnosis of attention disorder and four boys were treated with Ritalin, Atomoxetin. The two diagnostic groups did not differ regarding their non-verbal intelligence (AS: *M* = 117.3 ± 6.4, TD: *M* = 115.4 ± 9.2; *t*(28) = 0.646, *p* = 0.523), as assessed by the age-appropriate form of the German adaptation of the Culture Fair Intelligence Test (CFT) in the initial study (CFT1 [7]; CFT2 [8]). The study was in accordance with the 1964 Declaration of Helsinki and was approved by the authorized Ethics Committee. Informed written consent was obtained from parents of all participants prior to participation.

## Mittenecker pointing test (MPT)

In line with the procedures of the initial study, all participants were tested individually. They were introduced to the MPT by a child psychologist and were given some practice trials to ensure task comprehension. The MPT is a computer-based test that instructs participants to press (with their index finger) the keys of a keyboard with nine unlabelled keys irregularly distributed over the board in the most random or chaotic order possible, requiring 180 responses in total (for more details concerning the task please see [5]). The responses were paced by an acoustic signal (1.2/s) to control the rate of production.

As outcome variables we used two quantitative measures of deviation from randomness, namely Symbol Redundancy (SR) and Context Redundancy (CR). SR taps the memory component (memory monitoring/updating) of random sequence generation [9, 10]. A SR score of zero denotes maximal equality of the relative frequencies of chosen keys and, thus minimal predictability (best possible performance), whereas a (theoretical) score of 1.0 denotes maximal redundancy and, thus a complete lack of randomness.

CR examines the inhibition of prepotent response sequences and is based on the sequential probability of each chosen key. In true random series all possible dyads (pairs of adjacent responses) are approximately equiprobable, whereas their frequencies deviate from equality if responses are continuously influenced by previously chosen alternatives. The major part of the interindividual variance in CR is due to the tendency to repeat certain response sequences en bloc [11]. Hence, CR reflects the inhibition of developing routines [9]. A CR score of zero denotes the complete absence of any regular pattern while a score of 1.0 denotes the presence of a fixed, repetitive response pattern (i.e., maximal perseveration). For detailed information on the test and how to compute SR and CR, see [10].

## Results

**Fig. 1:**
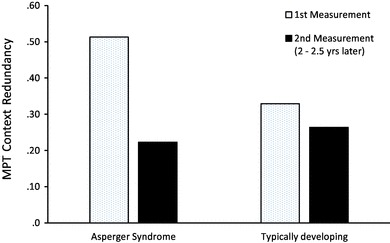
Mean MPT context redundancy (CR) scores in boys with Asperger syndrome and typically developing boys at the first measurement and at the second measurement 2–2.5 years later. Note: Higher CR scores indicate poorer inhibition of developing routines. Significant interaction effect of measurement (1st, 2nd) by diagnosis (AS, TD). Statistically significant difference between AS and TD controls at the first measurement only. Significant improvement in AS boys from first to second measurement

The hypothesis that younger AS children catch up with their TD counterparts when growing older was tested using an analysis of variance with measurement (first vs. second) as a within-subjects variable, diagnosis (AS vs. TD) as a dichotomous between-subjects variable, age at first measurement as a continuous between-subjects variable, and inhibition of developing routines (CR) as the dependent variable. Most relevant for the research question are the two-way interaction effect of measurement by diagnosis and the three-way interaction effect of measurement by diagnosis by age, which were both significant (*F*(1,26) = 15.4, *p* = 0.001, *η*_p_^2^ = 0.37 and *F*(2,26) = 6.2, *p* = 0.006, *η*_p_^2^ = 0.32, respectively). Cell means for the two-way interaction are depicted in Fig. 1. In AS boys, inhibition of prepotent responses, which was at a relatively poor level at the first measurement, significantly improved within the 2.5 years observation period (*t*(14) = 4.5, *p* = 0.001), with the effect that the marked average difference between AS and TD boys at the first measurement (*t*(28) = 4.3, *p* < 0.001) was not present any more when they were older (2nd measurement, *t*(28) = 1.3, *p* = 0.210).

**Fig. 2:**
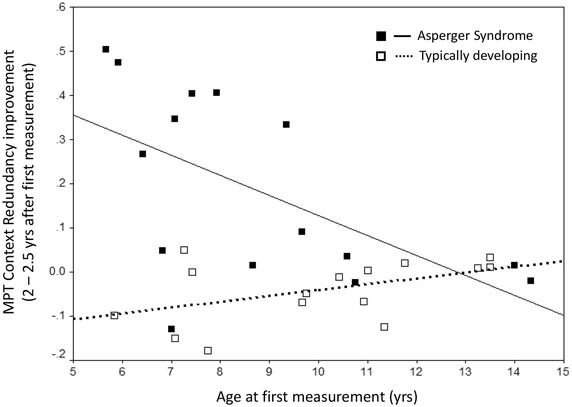
Moderating effect of age on improvements of MPT context redundancy (CR) within the 2–2.5 years interval in boys with Asperger syndrome vs. typically developing boys. Note: Higher positive values indicate greater improvements in the inhibition of developing routines. Significant three-way interaction effect of measurement (1st, 2nd) by diagnosis (AS, TD) by age (continuous variable). Depicted regression line (estimated improvements of CR in boys aged 5 to 15 years) significant in AS only

The significant three-way interaction effect indicated that the improvements in CR varied with the participants’ age at first measurement. To illustrate this interaction effect, Fig. 2 shows the raw improvements in CR scores for all participants (calculated as the difference between CR at the first minus CR at the second measurement), as well as the respective regression lines (estimated improvements of CR in boys aged 5 to 15 years). The regression line in AS boys was significant (*β* = − 0.58, *p* = 0.024). The regression line in TD boys was non-significant (*β* = 0.47, *p* = 0.078). The findings indicate more marked improvements within the 2.5 years interval in younger AS children, while older AS boys as well as TD boys remained at a relatively constant level throughout. Together, this further substantiates the notion that AS children (on average) catch up with TD children when they grow older as regards the basic inhibition of developing routine response patterns.

The main effects as well as the interaction effect of diagnosis by age were also significant in this analysis but are clearly attributed to the large higher-order interaction effects (diagnosis *F*(1,26) = 10.1, *p* = 0.004, *η*^2^ = 0.28; measurement *F*(1,26) = 4.5, *p* = 0.044, *η*^2^ = 0.15; diagnosis by age *F*(1,26) = 17.1, *p* < 0.001, *η*^2^ = 0.57). No significant effects were observed in an analogous analysis using the memory updating (SR) score as the dependent variable (diagnosis *F*(1,26) = 0.8, *p* = 0.394, *η*^2^ = 0.03; measurement *F*(1,26) = 1.8, *p* = 0.194, *η*^2^ = 0.06; measurement by diagnosis *F*(1,26) = 0.3, *p* = 0.577, *η*^2^ = 0.01; diagnosis by age *F*(1,26) = 1.4, *p* = 0.259, *η*^2^ = 0.10; measurement by diagnosis by age *F*(1,26) = 0.8, *p* = 0.469, *η*^2^ = 0.06).

## Discussion

The current longitudinal study complements and strengthens prior cross-sectional findings (for a review see [4]) by demonstrating that deficits in inhibition skills in children with ASD, i.e., the weak ability to inhibit or override the tendency to produce a dominant or prepotent but inappropriate response, become less marked with age, at least in cognitively able children with autism. More precisely, we found more marked improvements within a 2.5 year follow-up interval in younger AS children, while older AS boys as well as TD boys remained at a relatively constant level throughout. Similar findings of an age-moderating effect in prepotent inhibition tasks were shown in a meta-analysis, with younger individuals diagnosed with autism exhibiting poorer response inhibition in tasks such as the Go/No-Go test or the Stop signal test compared to adolescents and adults with ASD [12]. The dynamic nature of brain development in autism is also supported by some neuroimaging studies showing complex changes from childhood into adulthood in whole and regional brain volumes [13–15], patterns of functional connectivity [16], and white matter maturation [17].

In the present study, no group differences were found for the memory monitoring/updating component of random sequence generation. Previous research proposed typical and atypical developmental trajectories of distinct EFs in ASD with intact performance for prepotent response inhibition tasks, planning tasks and set-shifting tasks in older youths with ASD, but no age-moderating effect for spatial working memory or interference control tasks [12, 18, 19]. Additionally, several studies found an association between executive functions and adaptive behavior [20–25]. Compared with clinical psychometric tests of EFs, the MPT do not draw on academic skills that may vary considerably across different age-groups and may, therefore, better apt to disentangle more specific processes of EFs in juvenile AS (please see [5] for more details) and thus may be especially useful to study the association between executive functions and adaptive behavior in ASD with no intellectual deficits.

In summary, the current longitudinal study further substantiates the notion that AS children (on average) catch up with TD children when they grow older as regards the basic inhibition of developing routine response patterns.
